# Direct Inhibition of SARS-CoV-2 Spike Protein by Peracetic Acid

**DOI:** 10.3390/ijms24010020

**Published:** 2022-12-20

**Authors:** Yuichiro Yamamoto, Yoshio Nakano, Mana Murae, Yoshimi Shimizu, Shota Sakai, Motohiko Ogawa, Tomoharu Mizukami, Tetsuya Inoue, Taishi Onodera, Yoshimasa Takahashi, Takaji Wakita, Masayoshi Fukasawa, Satoru Miyazaki, Kohji Noguchi

**Affiliations:** 1 Laboratory of Molecular Targeted Therapy, Faculty of Pharmaceutical Sciences, Tokyo University of Science, 2641 Yamazaki, Noda 278-8510, Chiba, Japan; 2 Department of Pharmacy, Faculty of Pharmaceutical Sciences, Tokyo University of Science, 2641 Yamazaki, Noda 278-8510, Chiba, Japan; 3 Department of Biochemistry and Cell Biology, National Institute of Infectious Diseases, 1-23-1, Toyama, Shinjuku-ku, Tokyo 162-8640, Japan; 4 Department of Pharmaceutical Sciences, Teikyo Heisei University, 4-21-2, Nakano, Nakano-ku, Tokyo 164-8530, Japan; 5 Department of Virology I, National Institute of Infectious Diseases, 1-23-1, Toyama, Shinjuku-ku, Tokyo 162-8640, Japan; 6 Reseach Center for Drug and Vaccine Development, National Institute of Infectious Diseases, 1-23-1, Toyama, Shinjuku-ku, Tokyo 162-8640, Japan; 7 Department of Virology II, National Institute of Infectious Diseases, 1-23-1, Toyama, Shinjuku-ku, Tokyo 162-8640, Japan

**Keywords:** SARS-CoV-2, peracetic acid, spike protein, receptor-binding domain, ACE2

## Abstract

Peracetic acid (PAA) disinfectants are effective against a wide range of pathogenic microorganisms, including bacteria, fungi, and viruses. Several studies have shown the efficacy of PAA against severe acute respiratory syndrome coronavirus 2 (SARS-CoV-2); however, its efficacy in SARS-CoV-2 variants and the molecular mechanism of action of PAA against SARS-CoV-2 have not been investigated. SARS-CoV-2 infection depends on the recognition and binding of the cell receptor angiotensin-converting enzyme 2 (ACE2) via the receptor-binding domain (RBD) of the spike protein. Here, we demonstrated that PAA effectively suppressed pseudotyped virus infection in the Wuhan type and variants, including Delta and Omicron. Similarly, PAA reduced the authentic viral load of SARS-CoV-2. Computational analysis suggested that the hydroxyl radicals produced by PAA cleave the disulfide bridges in the RBD. Additionally, the PAA treatment decreased the abundance of the Wuhan- and variant-type spike proteins. Enzyme-linked immunosorbent assay showed direct inhibition of RBD-ACE2 interactions by PAA. In conclusion, the PAA treatment suppressed SARS-CoV-2 infection, which was dependent on the inhibition of the interaction between the spike RBD and ACE2 by inducing spike protein destabilization. Our findings provide evidence of a potent disinfection strategy against SARS-CoV-2.

## 1. Introduction

Toward the end of 2019, an outbreak of an unusual viral pneumonia caused by a new coronavirus, severe acute respiratory syndrome coronavirus 2 (SARS-CoV-2), occurred in Wuhan, China. Being highly transmissible, this novel coronavirus infection, also known as coronavirus disease 2019 (COVID-19), has spread rapidly around the world and become a serious threat to global public health [1,2,3].

Since the global pandemic caused by SARS-CoV-2, several studies on the environmental stability of SARS-CoV-2 have been reported. The stability of SARS-CoV-2 and severe acute respiratory syndrome coronavirus 1 (SARS-CoV-1) is similar under various experimental environments, such as plastic, stainless steel, and copper. SARS-CoV-2 and other human coronaviruses (HCoVs) have remarkably short persistence on copper and latex compared to other surfaces such as stainless steel, plastics, and glass [4,5]. In general, the stability of a particular virus in the environment is important for its spread. However, in addition to the virus characteristics, the characteristics of the biotic or abiotic environmental surface and the environmental conditions, particularly temperature and relative humidity, are important factors in determining the infectivity retention and extent and speed of the spread of the virus [4,6,7]. Therefore, when new viruses such as SARS-CoV-2 become widespread, their long-term persistence on the environmental surface and fomites is expected. Surface disinfection can reduce the potential risk of fomite-mediated transmission due to virus persistence on the environmental surface [8]. Fomite-mediated transmission is not the primary route of SARS-CoV-2 infection [9]. However, the high transmission rate of variants of concern (VOCs), such as the Omicron variant, raises questions and concerns regarding the enhanced environmental stability of the virus. To date, differences in the environmental stability of VOCs have not been reported consistently. The Alpha and Beta variants do not exhibit any difference in environmental stability on silver, copper, and stainless-steel discs [10]. However, the Beta variant was more thermostable than the other variants at physiological temperatures, which was correlated with plaque size [11]. Furthermore, the Alpha, Beta, Delta, and Omicron variants exhibited approximately two-fold longer survival times on plastic surfaces than the Wuhan strain. In particular, the Omicron variants (BA.1 and BA.2) have the longest survival times [12]. Considering the difference in environmental stability of SARS-CoV-2 variants, it is necessary to evaluate the effect of surface disinfection on the spread of these variants.

Various liquid disinfectants are routinely used to disinfect different surfaces in medical facilities [13,14]. The Centers for Disease Control and Prevention recommends the use of Environmental Protection Agency (EPA)-registered disinfectants for surface hygiene for COVID-19 patient care in healthcare settings. Several peracetic acid (PAA) formulations have been registered as COVID-19-specific disinfectants on EPA’s List N. PAA is a potent oxidant and microbicide; it can inactivate bacterial spores, fungi, and viruses [15,16,17]. In the medical field, it is widely used to disinfect endoscopes, sterilize bone allogeneic implants, and disinfect the surfaces of various medical equipment [18,19,20]. However, studies on the efficacy of PAA against coronaviruses are limited [21,22]; a previous study on SARS-CoV-2 has demonstrated the effects of dry fogging on the test surfaces of medical facilities that used PAA [14]. Furthermore, no previous study has investigated the mechanism underlying the disinfecting effect of PAA against SARS-CoV-2.

Viral infection is initiated by the binding of viral particles to host surface cellular receptors. In HCoVs such as SARS-CoV-1, the entry process is mediated by envelope-embedded, surface-located, spike glycoproteins [23]. The spike glycoprotein is a homotrimer, and each monomer contains two subunits: S1 and S2. The S1 subunit consists of the N-terminal domain and receptor-binding domain (RBD), and RBD recognizes and binds to the cellular receptor human angiotensin-converting enzyme 2 (hACE2) [24]. After the RBD binds to hACE2, the viral membrane fuses with the host cell membrane and the viral genome enters human cells to initiate the infection process [25,26,27]. Therefore, inhibiting the interaction between RBD and ACE2 can prevent SARS-CoV-2 infection, and RBD can be an effective target of disinfectants. The disinfecting effect of plasma-activated water is due to the inhibition of the RBD-ACE2 interaction via inactivation of the spike protein [28].

To date, there have been several reports showing the disinfecting effect of the PAA disinfectants against SARS-CoV-2; however, no study has demonstrated the disinfecting effect and mechanism against variant strains. In the present study, we examined the effects of PAA on the infectivity of SARS-CoV-2 variants and the alteration and function of the spike proteins.

## 2. Results

### 2.1. PAA Inhibited Pseudotyped SARS-CoV-2 Infectivity

**Figure 1:**
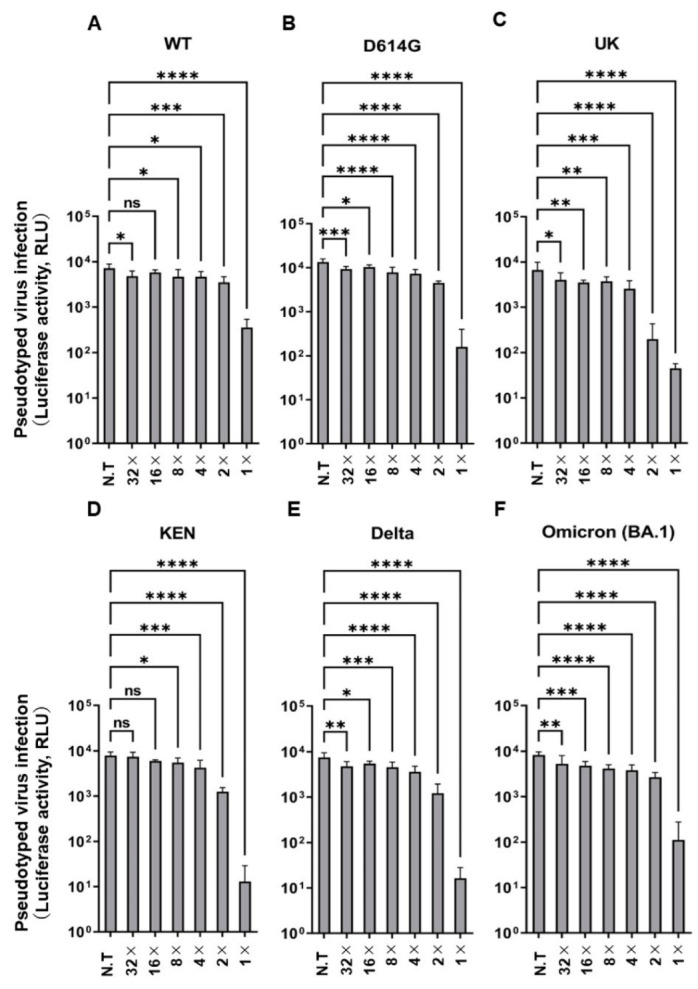
PAA impaired pseudotyped SARS-CoV-2 infection. Dose-dependent inactivation of pseudotyped SARS-CoV-2 by PAA in VeroE6/TMPRSS2 cells. The PAA dilution (1×) was prepared to a final concentration of 0.002%, followed by two-fold serial dilutions. (**A**) Wuhan-type (WT), (**B**) D614G, (**C**) Alpha (UK), (**D**) K417N/E484K/N501Y (KEN), (**E**) Delta, and (**F**) Omicron (BA.1). Experiments were independently repeated three times, and similar results were obtained. Data from sextuple samples are expressed as means ± SD. Statistical analysis was performed using one-way ANOVA and subsequent Dunnett’s test. * indicates *p* < 0.05, ** indicates *p* < 0.01, *** indicates *p* < 0.001, and **** indicates *p* < 0.0001, ns: not significant. N.T: no treatment.

We used a pseudotyped system with spike protein of SARS-CoV-2 to investigate the antiviral activity of PAA. At a final PAA concentration of 0.002%, the infectious activity of the Wuhan-type pseudotyped viruses was significantly suppressed, and the infectivity of variant strains was also suppressed (Figure 1A–F). Furthermore, we investigated the cytotoxicity at the concentration that inhibited infectivity. No cytotoxicity was observed in Vero E6/TMPRSS2 and HEK293T cells at these concentrations (Supplementary Figure S1A,B). This concentration was low, compared with the PAA concentrations used for disinfection of SARS-CoV-2 reported to date [14,29]. These results indicate that the pseudovirus infection was effectively inhibited by the PAA treatment at concentrations much lower than those of commonly used the PAA disinfectants.

### 2.2. Computational Analysis Showed That PAA Cleaves the Disulfide Bridges in the SARS-CoV-2 Spike Protein

**Figure 2:**
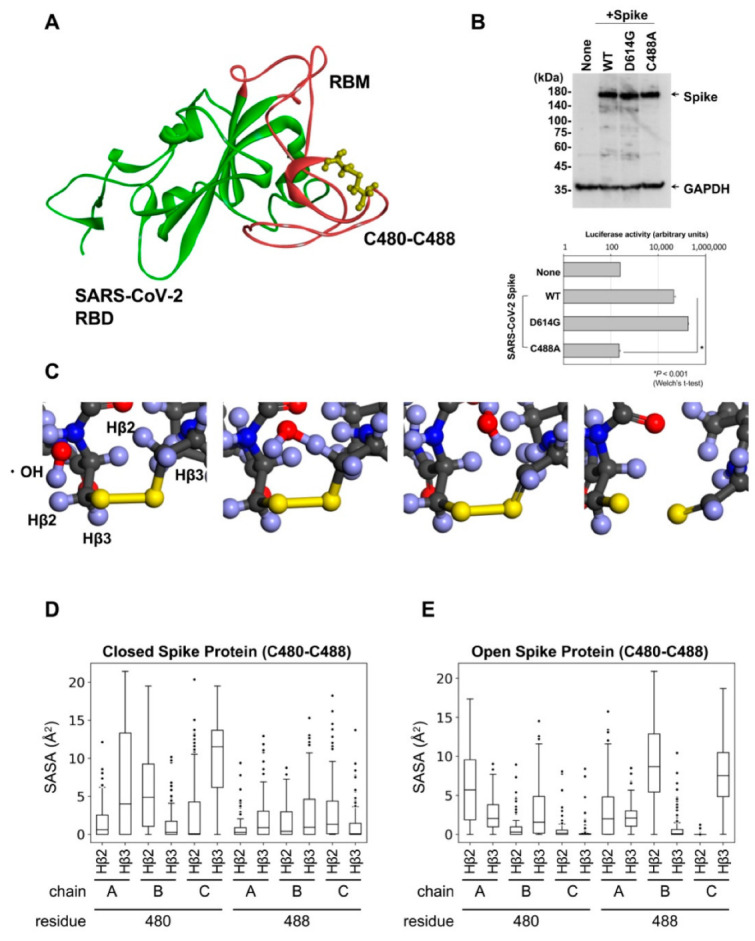
Disulfides bind with Cys480-Cys488 of SARS-CoV-2 spike RBM. (**A**) Three-dimensional structure of SARS-CoV-2 RBM colored in red. (**B**) Expression of Cys488A mutant spike proteins detected by Western blot analysis (upper panel). The infectivity of the pseudotyped viruses expressing each mutant spike protein were assessed by reporter luciferase activities in VeroE6/TMPRSS2 cells (lower panel). Data from triplicated samples were expressed as means ± SD. Statistical analysis was performed using Welch’s *t*-test. * indicates *p* < 0.001. (**C**) Using DFT calculation in Material Studio, hydroxyl radicals and six residues (Pro479-Cys480-Asn481, Asn487-Cys488-Tyr489) were reacted. (**D**,**E**) SASA (Å2) calculated on CβH atoms of each frame in MD simulation, which are next to disulfide-bonded sulfur.

The SARS-CoV-2 RBD contains four disulfide bridges, of which three disulfide pairs (Cys336-361, Cys379-432, and Cys391-525) help to stabilize the β sheet structure of the spike protein [26]. The remaining pair (Cys480-488) is located in the receptor-binding motif (RBM) (Figure 2A) and contributes to the interaction with ACE2 [26,30]. Next, we confirmed the expression of the spike protein in the Wuhan-type, D614G, and Cys488A mutants, and demonstrated that the Cys488A-mutant pseudotyped virus had reduced infectious activity (Figure 2B). PAA produces hydroxyl radicals and breaks the -SH and disulfide bridges in proteins [31]. However, it remains unclear whether the hydroxyl radicals disrupt the disulfide bridges in the RBD of the SARS-CoV-2 spike protein. Therefore, we investigated the reaction between the hydroxyl radicals and disulfide pairs in the RBD using computer analysis. We first obtained the three-dimensional structure of the RBD in both closed and open spike proteins using molecular dynamics (MD) simulation for 50 ns. Next, we performed density functional theory (DFT) using the resulting structure to investigate the reaction between the hydroxyl radicals and disulfide pairs in the RBM, which plays an important role in binding with ACE2. DFT calculations showed that the hydroxyl radicals reacted with the Hβ2 or Hβ3 atom, which is bonded with the β carbon (Cβ) next to the disulfide-bonded sulfur atom and was converted to water. Consequently, the bond between the Cβ atom and sulfur atom became a double bond, resulting in a cleaved disulfide bond (Figure 2C). The disulfide pairs in biological molecules react with the hydroxyl radicals [32], whereas Hβ2 or Hβ3 atom coordinates with the exterior of the three-dimensional structure, not the interior. To evaluate the ability of Hβ2 or Hβ3 to coordinate with hydroxyl radicals, we calculated the solvent accessible surface area (SASA), which indicates whether atoms are exposed to the solvent or hydroxyl radicals, using 100 structures obtained from MD simulation. In both open and closed states, the spike protein underwent a conformation in which Hβ atoms were exposed to the hydroxyl radicals (Figure 2D,E). These results suggest that the hydroxyl radicals produced by PAA cleave the disulfide bridges in the RBD.

### 2.3. PAA Led to the Destabilization of the SARS-CoV-2 Spike Protein

**Figure 3:**
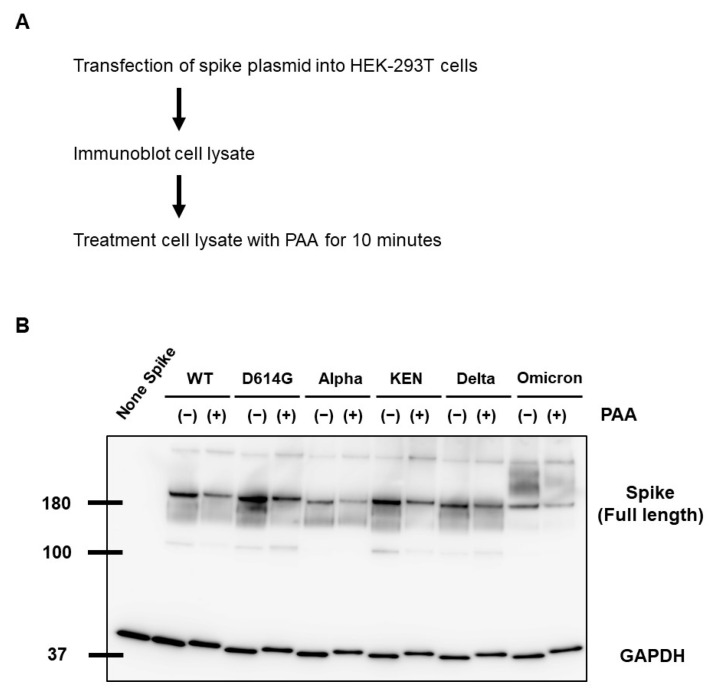
PAA led to SARS-CoV-2 spike protein destabilization. (**A**) WT and mutant spike proteins were expressed in HEK293T cells, and cell lysates were treated with PAA at a concentration of 0.01%. (**B**) Alteration of SARS-CoV-2 spike protein in PAA-treated cell lysates was detected by Western blot analysis. GAPDH is shown as a loading control. Experiments were repeated at least twice, and the representative data of the independent experiments are shown.

Reducing the disulfide stabilizer bridges of the SARS-CoV-2 spike protein induces the unfolding of recombinant spike protein and leads to spike protein destabilization [33]. Therefore, to determine whether the cleavage of the disulfide bridges affects spike protein destabilization, we assessed spike protein alteration in cell lysates following PAA treatment. The PAA treatment for 10 min at a final concentration of 0.01% led to spike protein alteration. Furthermore, this alteration was consistent in all the variant strains, including the Omicron variant (Figure 3A,B). Even with commercially available PAA disinfectants, the alteration of the spike protein was confirmed at the same concentration and processing time (Supplementary Figure S2A). On the other hand, SARS-CoV-2 nucleocapsid protein alteration was not confirmed by the PAA and commercial PAA disinfectant treatments (Supplementary Figure S2B). These results suggest that PAA selectively leads to the destabilization of the SARS-CoV-2 spike protein.

### 2.4. PAA Reduced the RBD-ACE2 Interaction

**Figure 4:**
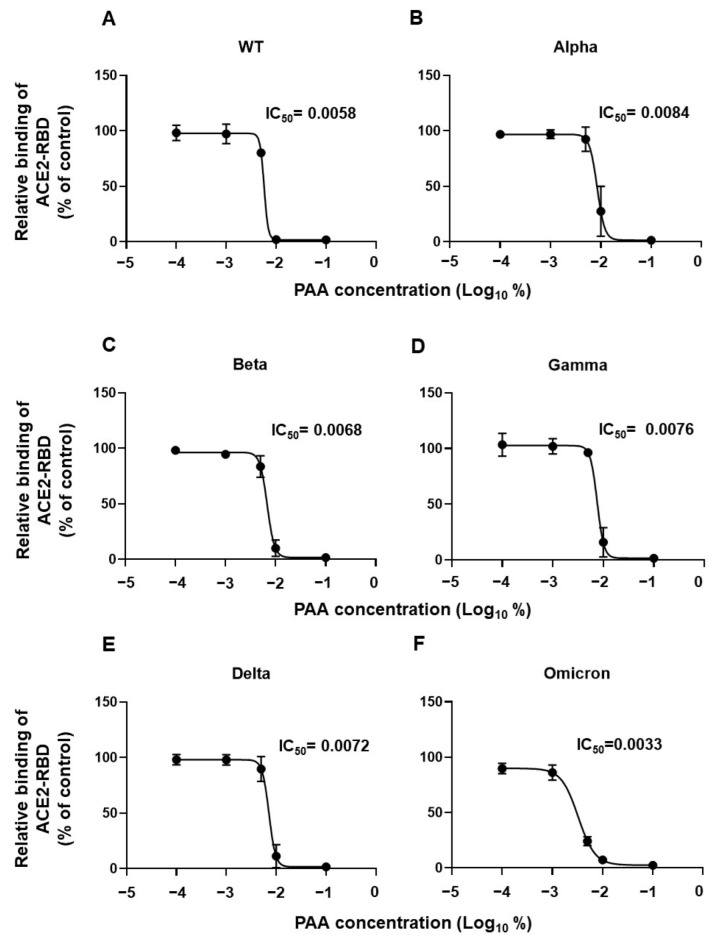
PAA impaired SARS-CoV-2 RBD interaction with ACE2 in vitro. Dose-dependent inhibition of recombinant RBD protein binding to ACE2 by PAA. The recombinant RBD protein was pretreated with PAA for 30 min, and its ACE2 binding was examined using ELISA. (**A**) WT, (**B**) Alpha, (**C**) Beta, (**D**) Gamma, (**E**) Delta, and (**F**) Omicron (BA.1). Experiments were independently repeated three times, and similar results were obtained. The 50% inhibitory concentration (IC50) was calculated using the GraphPad Prism software, version 9.4.1.

We demonstrated that PAA cleaves the disulfide bridges in the spike protein, leading to its alteration. Next, we investigated whether this effect on the spike protein affects the interaction between the RBD and ACE2. The binding ability of the RBDs of the Wuhan as well as the variant (Alpha, Beta, Gamma, Delta, Omicron) strains to ACE2 was markedly reduced with the 0.01% and 0.1% PAA treatment compared with that of untreated strains (Figure 4A–F). Furthermore, the IC50 value was highest in the Alpha variant and lowest in the Omicron variant, having a difference of approximately 2-folds. Similar results were obtained using commercially available PAA disinfectants (Supplementary Figure S3A–F). The effect was observed at a PAA concentration lower than the concentration used in a general PAA disinfectant. These results suggest that PAA reduces the infectivity of SARS-CoV2 by blocking the RBD-ACE2 interaction.

### 2.5. PAA Reduced Authentic Viral Load of SARS-CoV-2

**Figure 5:**
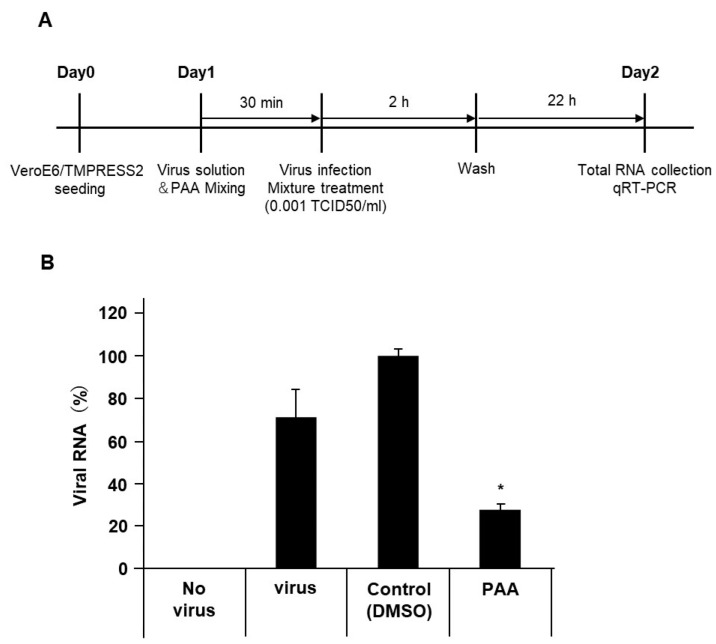
PAA reduced the SARS-CoV-2 viral load. The authentic SARS-CoV-2 solution was pretreated with PAA (0.0018%) for 30 min. After infection of VeroE6/TMPRSS2 cells, SARS-CoV-2 viral loads were measured using RT-qPCR. (**A**) Schematic diagram illustrating the experimental design. (**B**) Reduction in authentic SARS-CoV-2 viral load by PAA treatment. Data from triplicate samples are expressed as means ± SD. Statistical analysis was performed using one-way ANOVA and subsequent Dunnett’s test. * indicates *p* < 0.05.

**Figure 6:**
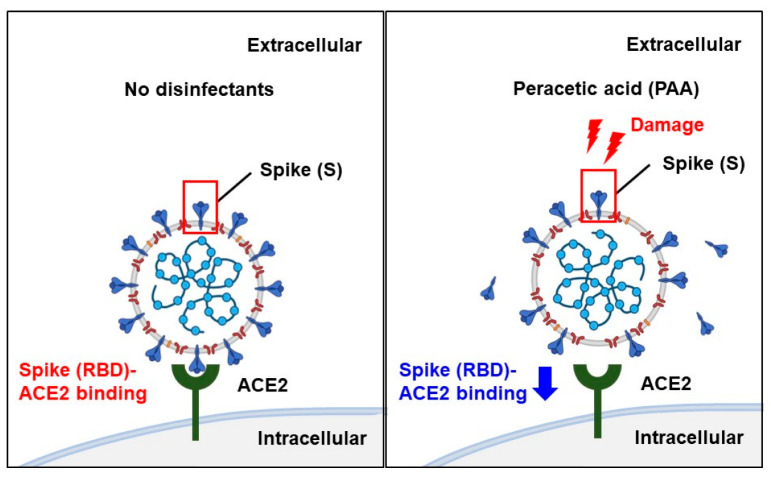
Scheme of disinfection effects of PAA against SARS-CoV-2. PAA is effective against a wide range of pathogens; it generates hydroxyl radicals, which cleave the Cys480-488 disulfide pair in the SARS-CoV-2 spike RBD and break the disulfide bond in the spike protein. As a result, it reduces the abundance of intact spike proteins and reduces the binding of the SARS-CoV-2 spike RBD to its host cell receptor, ACE2. PAA exhibits disinfection effects by inhibiting SARS-CoV-2 spike protein-mediated entry into the host cells. Schemas were created using BioRender.com.

Although spike, envelope, and membrane proteins together envelop the SARS-CoV-2 virion, most of the reported pseudotyped viruses are composed exclusively of spike protein. The presence of envelope and membrane proteins increases the virion infectivity by promoting the spike protein priming [34]. In addition, inconsistent results between the authentic and pseudotyped viruses have been reported in studies on neutralizing antibodies [35]. Since the pseudotyped viruses containing only spike proteins have limitations, we assessed the antiviral effect of PAA on authentic virus isolates. Similar to the results of the pseudotyped viruses, 30 min of the PAA treatment at a final concentration of 0.0018% significantly reduced the amount of viral RNA (Figure 5A,B). This result indicates that PAA is an effective disinfectant against authentic as well as pseudotyped SARS-CoV-2. Collectively, Figure 6 summarizes possible models of PAA disinfection mechanisms against SARS-CoV-2. We suggest that PAA exerts its antiviral effect by inhibiting the SARS-CoV-2 spike protein-mediated entry into host cells.

## 3. Discussion

The COVID-19 pandemic has led to a rapid increase in the use of disinfectants worldwide to prevent microbial infections [36,37,38]. However, excessive use of disinfectants poses a threat to living organisms and the environment [39,40,41]. Studies providing disinfection information for selecting effective and safe disinfectants against this virus are essential. PAA is a considerably safe disinfectant with a wide range of effects against pathogenic microorganisms [15,16,17]. However, few studies have demonstrated its disinfecting effect against SARS-CoV-2, and none have investigated the molecular mechanism of this effect. The present study examining the disinfecting effect of PAA against SARS-CoV-2 is the first to confirm that PAA affects this virus, including its variants, and demonstrate the molecular mechanism of its effect.

In the present study, PAA at a final concentration of 0.002% inhibited the infectious activity of the pseudotyped viruses, including variants, by ≥2 log10. This concentration was considerably lower than the concentration of PAA usually used in disinfectants. PAA is substantially less stable than hydrogen peroxide. A 40% PAA solution loses 1–2% of active ingredients per month, whereas 30–90% hydrogen peroxide solutions lose less than 1% of active ingredients per year. Diluted PAA solutions are more unstable, with 1% solution losing half its strength by hydrolysis in 6 days [42]. Proper use of surface disinfectants is crucial for preventing nosocomial infections [43,44]. PAA-based disinfectants are widely used in healthcare settings [18,19,20], but the use of PAA-based disinfectants containing low disinfectant levels has been reported to increase healthcare-associated *Clostridium difficile* infection [45]. Our results suggest that PAA has a disinfecting effect on SARS-CoV-2 at a low concentration of 0.002%. However, in healthcare settings, it should be used at an appropriate concentration, considering the stability of the PAA solution and the decrease in disinfectant levels.

In general, oxidizing compounds, such as PAA, oxidize the thiol groups (-SH) in cysteine residues, which form the disulfide bridges. As cysteine residues are located in the active sites of many bacterial enzymes, their oxidation by oxidizing compounds leads to the inactivation of these enzymes [46]. Reactivity with specific viral components can be used to identify the most sensitive regions of viral particles during disinfection [47], and PAA has been shown to be highly reactive with sulfur-containing amino acids such as cysteine [46,48]. In the present study, computational analysis demonstrated that PAA treatment cleaves the disulfide bridges in the RBD of the SARS-CoV-2 spike protein, and in vitro analysis revealed alterations in the spike protein and inhibition of the RBD-ACE2 interaction. These findings confirm the assumption that PAA exhibits a disinfecting effect by disrupting the -SH and disulfide bridges in the SARS-CoV-2 spike protein. Therefore, we infer that PAA induces damage to spike proteins by disrupting the disulfide bridges in RBDs and reduces their abilities to interact with the hACE2 receptor to initiate viral entry and infection.

Interestingly, the IC50 value of PAA was lowest in the Omicron variant. The affinity of RBD-ACE2 is higher in the Alpha, Beta, and Gamma variants than in the Delta and Omicron variants [49]. Moreover, the Omicron variant has a lower affinity than the Delta variant [50,51], as demonstrated by surface plasmon resonance analysis, MD simulations, and ELISA bioassays. The differences in RBD-ACE2 affinity between variants might contribute to the differences in the inhibition of interactions with PAA treatment. The stability of Omicron BA.1 and BA.2 has been reported to be higher than that of other variants in alcohol-based disinfection [12]. This study suggests that PAA might be a disinfectant that should be used more selectively for non-biological surface disinfection in Omicron variant epidemics.

In the present study, we demonstrated that PAA has a disinfecting effect even on authentic viruses. Several studies have reported an association between diarrhea and gastroenteritis symptoms and infections by HCoVs [52,53], suggesting potential foodborne and waterborne transmission of SARS-CoV-2. Furthermore, recent studies have shown that SARS-CoV-2 can survive in wastewater and has a decay half-life of 0.49 day at ambient temperature [54,55]. Although these previous studies suggest possible transmission of SARS-CoV-2 through the water environment, the application of conventional disinfectants at high dosages may generate toxic residual by-products in the water environments. However, PAA rarely forms toxic disinfection by-products and is widely used as a disinfectant in water environments as an alternative to chlorine-based disinfectants [42,56]. Our results suggest that PAA is a safe and effective disinfectant for water environments during the SARS-CoV-2 epidemic. To verify the effectiveness of disinfectants in SARS-CoV-2 contaminated water, it makes it imperative to use infectious SARS-CoV-2 virus rather than viral RNA, although this would involve a high level of safety to conduct.

This study has a primary limitation. The purpose of this study was to verify the effect of PAA against SARS-CoV-2, including variant strains, and the mechanism of its effect. Thus, it has been limited to investigation under an experimental environment. It has not been verified whether the same mechanism exerts a disinfection effect in an environment with many impurities. Therefore, further studies are necessary to determine its efficacy in a range of environments and situations.

In conclusion, PAA inhibited SARS-CoV-2 infection through a mechanism that cleaves the disulfide bridges in the RBD, induces spike protein dysfunction, and subsequently inhibits the interaction of the RBD with ACE2 (Figure 6). Furthermore, in the RBD-ACE2 binding assay, Omicron variants were most sensitive to PAA and showed lower IC50 values than the other variants. PAA as a disinfectant may be selectively used for epidemics of Omicron variants. These novel findings provide evidence of a potent disinfection strategy against SARS-CoV-2.

## 4. Materials and Methods

### 4.1. Cells and Reagents

HEK293T and VeroE6/TMPRSS2 (JCRB, JCRB1819) cells were cultured in Dulbecco’s modified Eagle’s medium (DMEM) supplemented with 7.5% (*v*/*v*) fetal bovine serum (FBS) and kanamycin (50 µg/mL) in 5% CO_2_ and 95% air at 37 °C. PAA solution and commercial PAA disinfectants were purchased from Sigma-Aldrich (Sigma-Aldrich, Tokyo, Japan) and Saraya (Saraya Co., Ltd., Osaka, Japan), respectively. PAA and commercial PAA disinfectants were dissolved at indicated concentrations in DMEM supplemented with 1% (*v*/*v*) FBS.

### 4.2. Preparation of Pseudotyped SARS-CoV-2

Pseudotyped SARS-CoV-2 was prepared as previously described [57]. Briefly, the plasmid pUC57-2019-nCoV-S (Human), containing synthetic cDNA to express SARS-CoV-2 spike protein with human codon optimization, was purchased from GenScript Japan Inc. (Tokyo, Japan) and cloned into the expression plasmid pcDNA3.1. Mutant spike cDNAs were synthesized using GenScript. The plasmids used in this study are listed in Supplementary Table S1. For retrovirus-based pseudotyped virus production, HEK293T cells were co-transfected with spike-expressing plasmids containing phCMV-Gag-Pol 5349 and reporter pTG-Luc126 plasmids using the PEIpro^®^ transfection reagent (Polyplus Transfection, NY, USA). Briefly, 2 × 10^6^ 293T cells were seeded in a T-25 flask on day 1, and the cells were co-transfected following the manufacturer’s instructions on day 2. On day 3, the growth medium was added to the flask for an additional two days of culture. The cell supernatant containing pseudotyped virus was collected, filtered through a 0.45 μm filter, and aliquoted to be stored at −80 °C.

### 4.3. Luciferase Assay for Pseudotyped Virus Infection

VeroE6/TMPRSS2 cells were seeded in a 96-well white plate at a density of 2–2.5 × 10^4^ cells per well and cultured for 24 h. After culturing for 24 h, pseudotyped viruses were added to each well and cultured for three days. For PAA pretreatment, the virus was preincubated in a medium containing PAA at the indicated concentration (DMEM containing 1% FBS) for 10 min at room temperature and then added to the wells. After three days, the medium was removed. Cells were washed once with phosphate-buffered saline (PBS) and subsequently lysed using a luciferase assay reagent (PicaGene MelioraStar-LT Luminescence Reagent, TOYO B-NET Co., Ltd., Tokyo, Japan). Luminescence signals were measured using an EnVision multilabel plate reader (PerkinElmer, 2104-0020, Waltham, MA, USA).

### 4.4. Cell Counting Kit-8 (CCK-8) Assay

We assessed the cytotoxic effect of PAA on HEK293T and VeroE6/TMPRSS2 cells using cell counting kit-8 (CCK-8) assay. Cytotoxicity was assessed at concentrations similar to those used in the luciferase assay for pseudotyped virus infection. To confirm the cytotoxicity of the disinfectants, the virus solution was not added. Briefly, HEK293T and VeroE6/TMPRSS2 cells were seeded in 96-well plates at a density of 2 × 10^4^ cells/well and cultured in the above complete medium for 24 h. Thereafter, PAA was added at the indicated concentration, and the cells were cultured for three days. Then, we added 10 μL CCK-8 solution (Dojindo Laboratories, Kumamoto, Japan) to the cells and incubated them for 3 h at 37 °C. A multimode microplate reader (SpectraMax iD3; Molecular Devices, San Jose, CA, USA) was used to detect the absorbance at 450 nm.

### 4.5. Computational Analysis

The three-dimensional structures of spike protein were obtained from the closed state (PDBID: 6VXX, https://www.rcsb.org/structure/6vxx, accessed on 17 February 2022) and open state (PDBID 6VYB, https://www.rcsb.org/structure/6VYB, accessed on 17 February 2022) downloaded from RSCB PDB (https://www.rcsb.org/) [24]. Both models had 27–1147 residues, but some loop regions and disulfide bridges were missing. Missing amino acids were compensated using Discovery Studio (Dassault Systemes Co., Ltd., Velizy-Villacoublay, France). Next, all-atom simulations were run using Amber16 (https://ambermd.org/), and the parameter sets were modeled in the ff14SB force field. Each structure was placed in a periodic box of TIP3P water with an 8 Å solvent buffer between the spike protein and edge of the box. Energy minimization was performed under a constant volume in 10,000 steps, consisting of 2000 steepest descent and 8000 conjugate gradient. Heating was performed from 0 to 310 K (0.7 ns) and maintained at 310 K (0.3 ns). Equilibration was performed using NPT ensemble for 2 ns. The cutoff distance for non-bonded pair interactions was 10 Å. Finally, production simulation was obtained for 50 ns trajectories every 0.5 ns under 310 K and one atom. The reaction between hydroxyl radicals and Cys480-Cys488 in the spike protein was conducted using DFT calculations in Material Studio (Dassault Systemes Co., Ltd., Velizy-Villacoublay, France). DFT calculates the quantum mechanics of the internal energy of molecules and predicts the reaction. Spike protein was obtained by MD simulation, and six residues (Pro479-Cys480-Asn481, Asn487-Cys488-Tyr489) were extracted from a 3 Å region in the center of Cys480-Cys488. The six residues and hydroxyl radicals were set and reacted using DFT calculations.

### 4.6. Western Blot Analysis

HEK293T cells were seeded in a 12-well plate at a density of 2 × 10^5^ cells/well and cultured for 24 h. Next, the cells were transiently transfected with each spike plasmid and cultured for two days. Thereafter, the cells were lysed using ULTRARIPA A buffer (BioDynamics Laboratory Inc., Tokyo, Japan). Cell lysates were treated with PAA at the indicated concentration for 10 min. Then, the amount of protein in the cell lysates was measured using BCA protein assay kit (Takara Bio Inc., Shiga, Japan). Equal amount of protein was determined by SDS-PAGE and electrotransferred to an Immobilon-P PVDF membrane (EMD Millipore, Billerica, MA, USA). Western blotting was performed using the standard method, and the following antibodies were used: anti-SARS-CoV-2 spike (1A9, GTX632604, mouse monoclonal antibody, GeneTex, Irvine, CA, USA), anti-SARS-CoV-2 nucleocapsid (HL344, GTX635679, rabbit monoclonal antibody, GeneTex), anti-GAPDH (3H12, M171-3, mouse monoclonal antibody, MBL), and horseradish peroxidase-conjugated sheep anti-mouse IgG (NA931, Amersham Biosciences, Amersham, UK). Immunoblot signals were developed using EzWestLumi plus (ATTO Corp., Tokyo, Japan) and recorded with an ImageQuant LAS4000 mini image analyzer (GE Healthcare, Tokyo, Japan).

### 4.7. Preparation of Recombinant RBD and Soluble ACE2

The expression vector encoding the RBD of the Wuhan type and mutants SARS-CoV-2 spike protein was constructed as previously described [57]. The plasmids of SARS-CoV-2 spike protein RBD mutants were prepared by site-directed mutagenesis using the KOD-Plus-Mutagenesis kit (Toyobo, Osaka, Japan). A gene encoding human soluble ACE2 (sACE2, GenBank accession number NM_001371415.1, residues 18–614 aa) fused to a C-terminal Fc tag was cloned into the pSecTag2 vector (Thermo Fisher Scientific) between the Ig kappa signal peptide and stop codon. Recombinant proteins were produced using FreeStyle 293-F cells, according to the manufacturer’s instructions (Thermo Fisher Scientific).

### 4.8. ELISA for In Vitro Binding Assay

We coated 96-well EIA/RIA plates (Corning-Coaster, Tokyo, Japan) with 100 μL of 1 µg/mL human sACE2-Fc protein overnight at 4 °C. The plates were washed with PBS containing 0.1% Tween 20 and blocked with 100 μL of 1% bovine serum albumin (BSA) (fatty acid-free, Fujifilm Wako Pure Chemical, Osaka, Japan) in PBS for 2 h at room temperature. SARS-CoV-2 RBD peptides (100 ng/well) were preincubated with 100 μL of 1% BSA in PBS at the indicated concentrations of PAA for 30 min at room temperature and then added to the wells. After incubation for 2 h at room temperature, the plates were washed with PBS/Tween and treated with 200 μL PBS/BSA containing mouse Avi-tag monoclonal antibody (GenScript, Tokyo, Japan, A01738, 1:5000 dilution) for 1 h at room temperature. After washing with PBS/Tween, the plates were further incubated with 100 μL PBS/BSA containing HRP-conjugated anti-mouse IgG (1:5000 dilution, Jackson ImmunoResearch Laboratory Inc., West Grove, PA, USA) for 1 h at room temperature. Bound RBD proteins were detected by adding 100 μL TMB substrate solution (Thermo Fisher Scientific, Waltham, MA, USA). The reaction was stopped by the addition of 50 μL of 2 M H_2_SO_4_. Optical density was measured at 450 nm using a multimode microplate reader (SpectraMax iD3; Molecular Devices).

### 4.9. SARS-CoV-2 Viral RNA Extraction and qRT-PCR

VeroE6/TMPRSS2 cells were seeded in 48-well clear plate at a density of 5.0 × 10^4^ cells per well and cultured for 24 h. After culturing for 24 h, the cells were inoculated with 0.001 TCID 50/cell of SARS-CoV-2 JPN/TY/WK-521 strain and incubated at 37 °C in DMEM supplemented with 2% FBS for 2 h. For PAA pretreatment, virus was preincubated with 2% FBS medium containing PAA at the indicated concentration for 30 min at 37 °C, and then added to well. After 2 h, PAA and virus mixture was removed. Cells were washed three times with 10% FBS containing medium and subsequently cultured for 22 h. Viral RNA was extracted from same aliquots of cell suspension using the Blood/Cultured cell total RNA kit (Favorgen Biotech Corporation, Pingtung, Taiwan). Real-time qPCR was performed by using THUNDERBIRD probe one-step qRT-PCR kit (Toyobo Co., Ltd., Osaka, Japan) in a Light Cycler 96 (Roche Diagnostics, Basel, Switzerland) with the virus specific primers and a TaqMan probe suitable for the detection of SARS-CoV-2 designed by NIID (N2 set, Eurofins Genomics, Tokyo, Japan).

### 4.10. Statistical Analysis

Statistical analyses were performed using the GraphPad Prism software (version 9.4.1). Data were expressed as mean and standard deviation (SD). Differences between the means were evaluated using one-way ANOVA and Dunnett’s method for multiple comparisons. Differences between groups were considered significant if *p* < 0.05.

## Data Availability

Not applicable.

## Supplementary Materials

The following supporting information can be downloaded at: https://www.mdpi.com/article/10.3390/ijms24010020/s1.
