# Supplementary material for: Direct Inhibition of SARS-CoV-2 Spike Protein by Peracetic Acid
Source: Int J Mol Sci. 2022 Dec 20;24(1):20. doi: 10.3390/ijms24010020 (PMC9820423; doi:10.3390/ijms24010020)
Supplement: Supplementary file 1 [file ijms-24-00020-s001.zip › Supporting Table.pdf]

Supporting Table

Supplementary Table S1. SARS-CoV-2 plasmids in this study.

**Plasmid names**	**Protein sequence and mutation**
pcDNA3.1-spike/WT	Wuhan Hu-1 spike, YP_009724390.1
pcDNA3.1-N	pcDNA3.1 SARS-CoV-2 N was a gift from Jeremy Luban (Addgene plasmid, # 158079 ; http://n2t.net/addgene:158079; RRID:Addgene_158079)
pcDNA3.1-spike/D614G	D614G
pcDNA3.1-spike/C488A	C488A
pcDNA3.1-spike/alpha	H69/V70-del, Y144-del, N501Y, A570D, D614G, P681H, T716I, S982A, D1118H
pcDNA3.1-spike/KEN	K417N/E484K/N501Y
pcDNA3.1-spike/delta	L5F, T19R, E156G, F157/R158-del, L452R, T478K, D614G, P681R, D950N
pcDNA3.1-spike/omicron (BA.1)	A67V, H69/V70-del, T95I, G142D, del143-145, ins214EPE, NL211-212I, G339D, S371L, S373P, S375F, K417N, N440K, G446S, S477N, T478K, E484A, Q493R, G496S, Q498R, N501Y, Y505H, T547K, D614G, H655Y, N679K, P681H, N764K, D796Y, N856K, Q954H, N969K, L981F
pCAGGS_MCS-CoV2-RBD(331-529)-6xHis-Avitag_WT	Wuhan Hu-1 spike, YP_009724390.1, amino acids 331-529.
pCAGGS_MCS-CoV2-RBD(331-529)-6xHis-Avitag_alpha	N501Y
pCAGGS_MCS-CoV2-RBD(331-529)-6xHis-Avitag_beta	K417N/E484K/N501Y
pCAGGS_MCS-CoV2-RBD(331-529)-6xHis-Avitag_gamma	K417T/E484K/N501Y
pCAGGS_MCS-CoV2-RBD(331-529)-6xHis-Avitag_delta	L452R
pCAGGS_MCS-CoV2-RBD(331-529)-6xHis-Avitag_omicron (BA.1)	G339D, S371L, S373P, S375F, K417N, N440K, G446S, S477N, T478K, E484A, Q493R, G496S, Q498R, N501Y, Y505H
