# Supplementary figures and images for: Direct Inhibition of SARS-CoV-2 Spike Protein by Peracetic Acid
Source: Int J Mol Sci. 2022 Dec 20;24(1):20. doi: 10.3390/ijms24010020 (PMC9820423; doi:10.3390/ijms24010020)

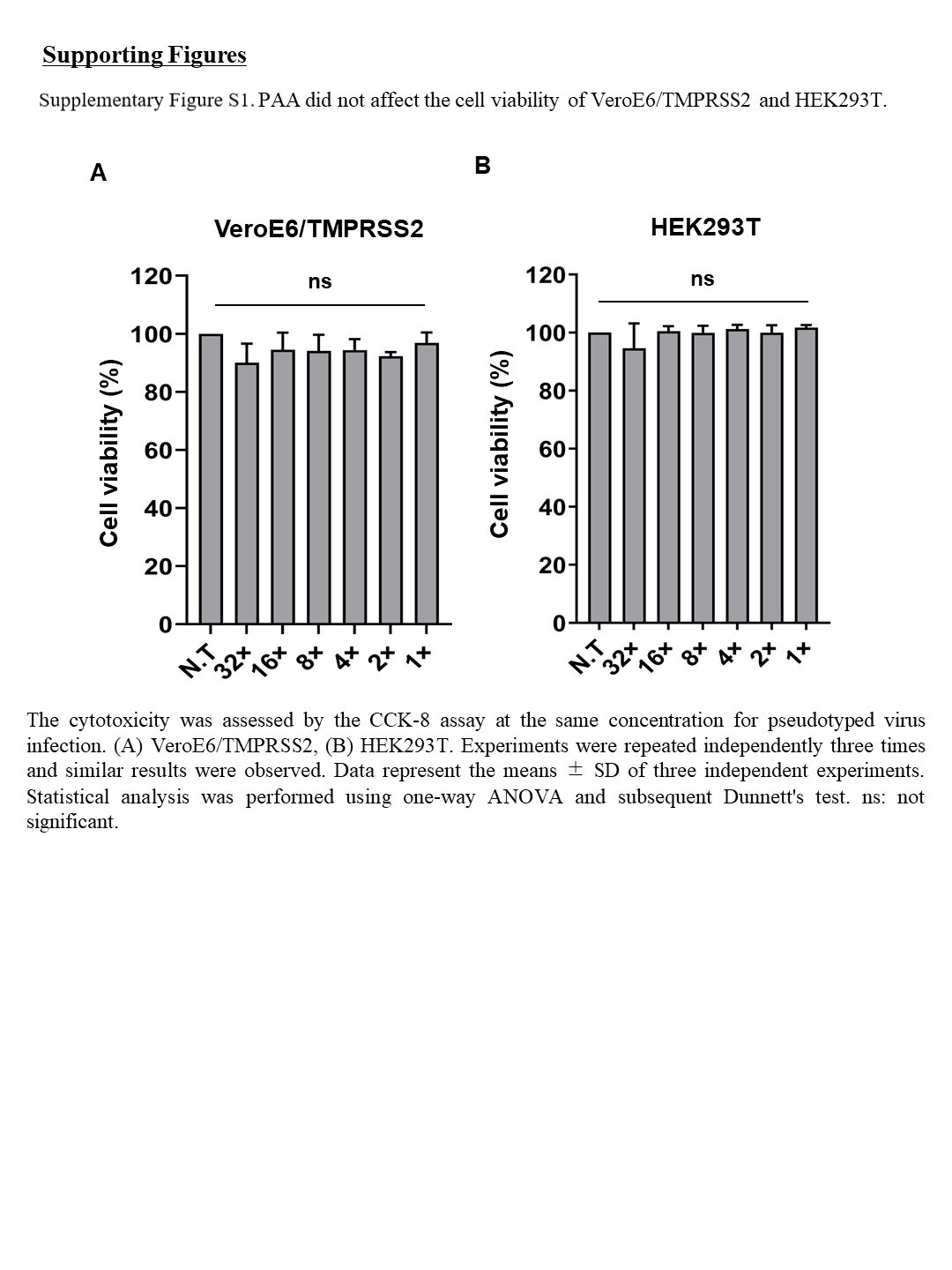

Supplement: Supplementary file 1 [file ijms-24-00020-s001.zip › Supplementary Figure S1.jpg]

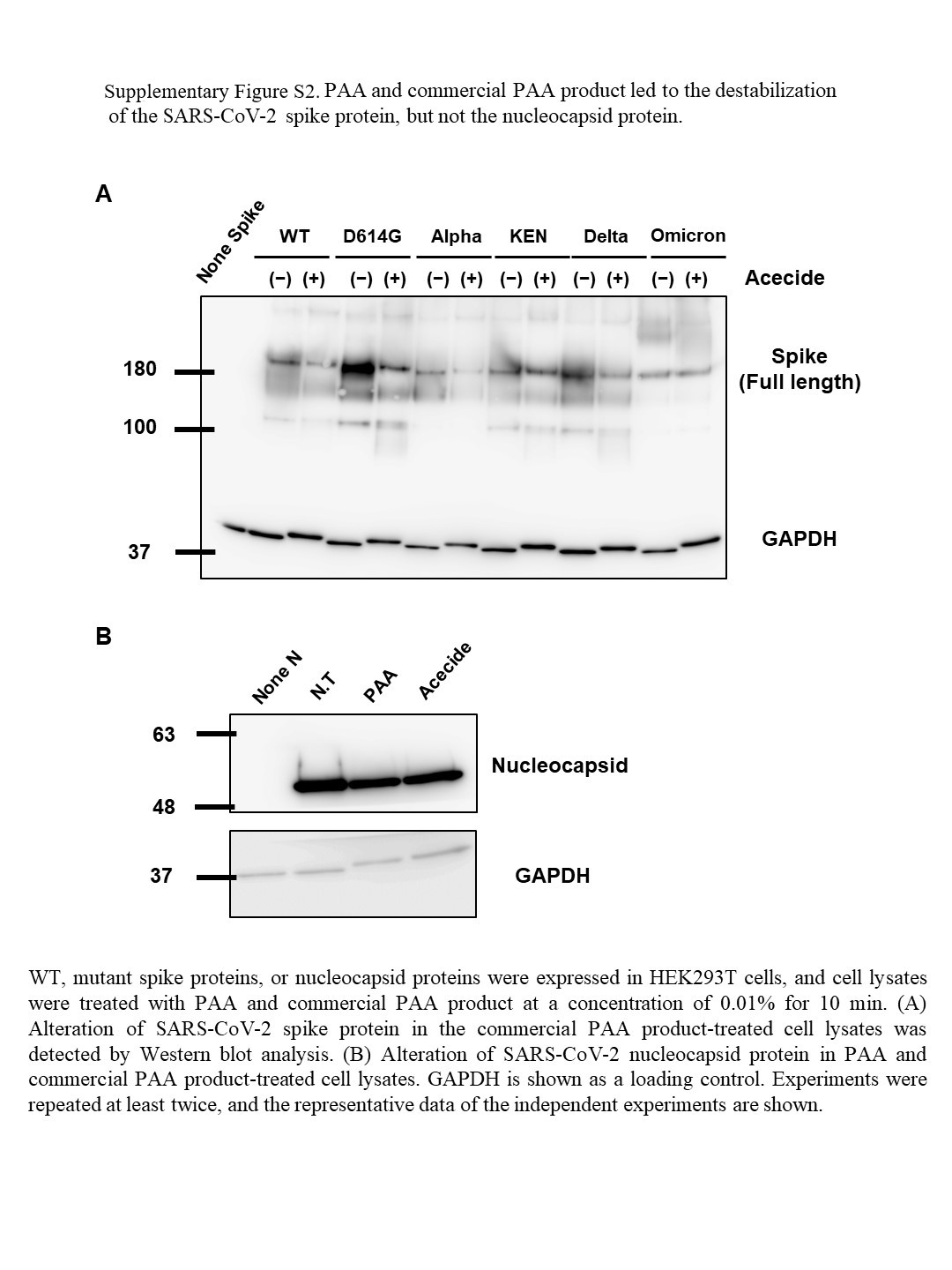

Supplement: Supplementary file 1 [file ijms-24-00020-s001.zip › Supplementary Figure S2.jpg]

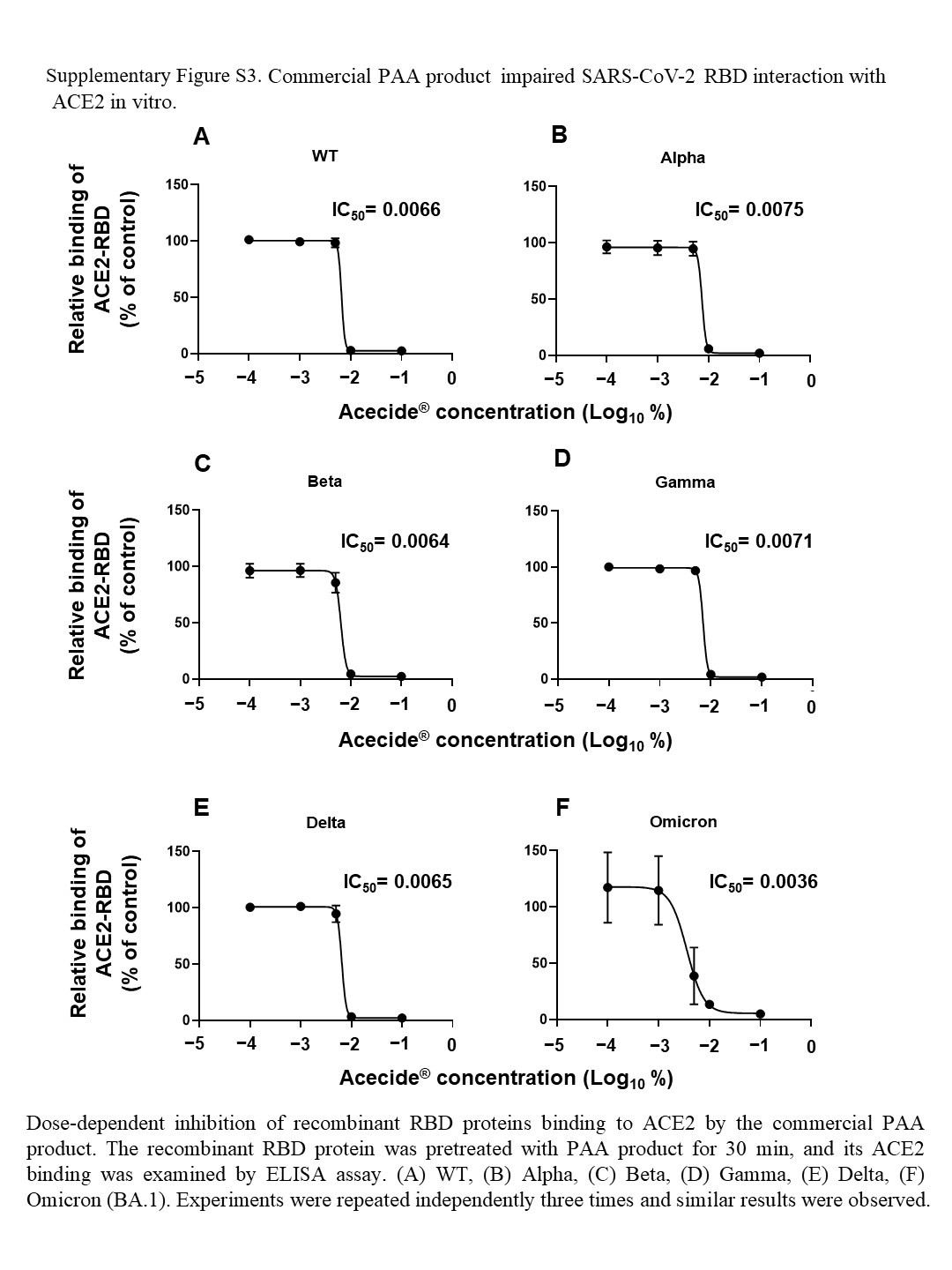

Supplement: Supplementary file 1 [file ijms-24-00020-s001.zip › Supplementary Figure S3.jpg]

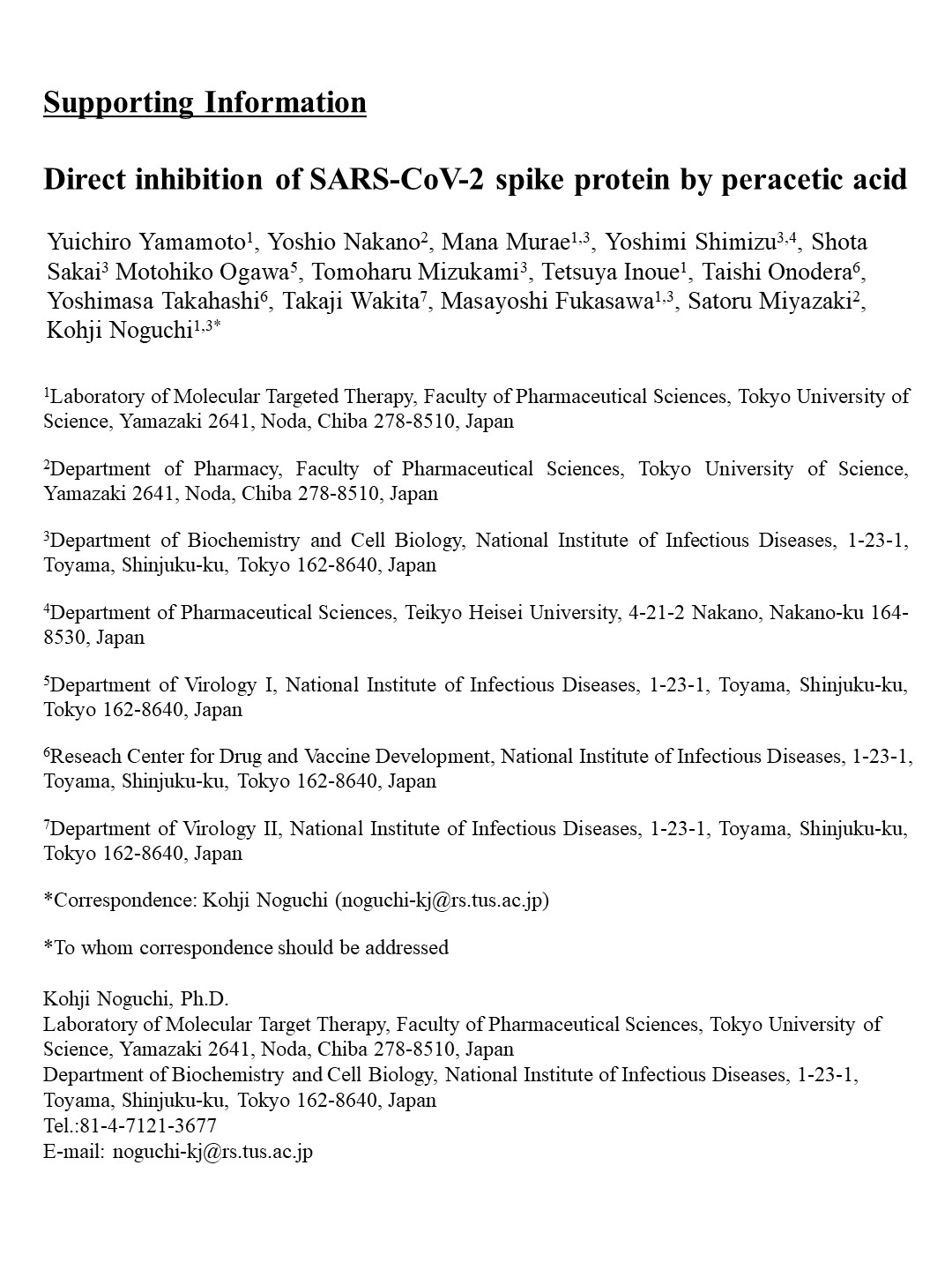

Supplement: Supplementary file 1 [file ijms-24-00020-s001.zip › Supplementary Materials.jpg]
